# Day-4 Myeloid Dendritic Cells Pulsed with Whole Tumor Lysate Are Highly Immunogenic and Elicit Potent Anti-Tumor Responses

**DOI:** 10.1371/journal.pone.0028732

**Published:** 2011-12-14

**Authors:** Cheryl Lai-Lai Chiang, Andrea R. Hagemann, Rachel Leskowitz, Rosemarie Mick, Thomas Garrabrant, Brian J. Czerniecki, Lana E. Kandalaft, Daniel J. Powell, George Coukos

**Affiliations:** 1 Ovarian Cancer Research Center, University of Pennsylvania, Philadelphia, Pennsylvania, United States of America; 2 Department of Biostatistics and Epidemiology, University of Pennsylvania, Philadelphia, Pennsylvania, United States of America; 3 Rena Rowan Breast Cancer Center, University of Pennsylvania, Philadelphia, Pennsylvania, United States of America; Tulane University, United States of America

## Abstract

“Day-7” myeloid DCs are commonly used in the clinic. However, there is a strong need to develop DCs faster that have the same potent immunostimulatory capacity as “Day-7” myeloid DCs and at the same time minimizing time, labor and cost of DC preparations. Although “2 days” DCs can elicit peptide-specific responses, they have not been demonstrated to engulf, process and present complex whole tumor lysates, which could be more convenient and personalized source of tumor antigens than defined peptides. In this preclinical study, we evaluated the T-cell stimulatory capacity of Day-2, Day-4, and Day-7 cultured monocyte-derived DCs loaded with SKOV3 cell whole lysate prepared by freeze-thaw or by UVB-irradiation followed by freeze-thaw, and matured with lipopolysaccharide (LPS) and interferon (IFN)-gamma. DCs were evaluated for antigen uptake, and following maturation with LPS and IFN-gamma, DCs were assessed for expression of CD80, CD40, CD86, ICAM-1 and CCR7, production of IL-12p70 and IP-10, and induction of tumor-specific T-cell responses. Day-4 and Day-7 DCs exhibited similar phagocytic abilities, which were superior to Day-2 DCs. Mature Day-7 DCs expressed the highest CD40 and ICAM-1, but mature Day-4 DCs produced the most IL-12p70 and IP-10. Importantly, Day-4 and Day-7 DCs derived from ovarian cancer patients stimulated equally strongly tumor-specific T-cell responses. This is the first study demonstrating the highly immunogenic and strong T-cell stimulatory properties of Day-4 myeloid DCs, and provided important preclinical data for rapid development of potent whole tumor lysate-loaded DC vaccines that are applicable to many tumor types.

## Introduction

Dendritic cells (DCs) are the most potent antigen-presenting cells in the human immune system. Due to their unique ability to prime and stimulate both CD8^+^ and CD4^+^ T cells, DCs loaded with whole tumor lysate have been investigated in several clinical trials for their ability to induce therapeutic anti-tumor T cell responses [1], [2], [3], [4], [5], [6], [7], [8]. Beneficial anti-tumor responses have been observed in some patients, illustrating the potential of this approach. DCs can be classified into different subsets depending on their lineage and receptor expression pattern. Their distinct biology can be exploited for different therapeutic strategies. The most widely used DCs for clinical trials are the myeloid DCs that are differentiated from peripheral blood monocytes in the presence of recombinant granulocyte-macrophage colony stimulating factor (GM-CSF) and interleukin 4 (IL-4). In most trials, 7 days are used to generate fully-differentiated “classic” DCs [9], [10], [11]. These DCs exhibit high phagocytic capability. Upon maturation with an appropriate stimulus, Day-7 DCs upregulate surface markers such as CD80, CD86, CD40, and migration markers such as CCR7, and can efficiently prime naive T cells [12], [13], [14].

To generate DC-based vaccines for rapid clinical trial use, shorter DC differentiation protocols have been investigated. Czerniecki and colleagues established a “rapid DC” protocol in which monocytes were exposed for 2 days to recombinant GM-CSF and IL-4, pulsed with an immunodominant HER-2/neu peptide, and subsequently matured with lipopolysaccharide (LPS) and interferon (IFN)-γ before vaccination of patients with ductal carcinoma *in situ* of the breast [15]. Such 2-day “rapid DCs” exhibit high surface expression of CD80, CD86, CD40, HLA-DR, MHC Class I, and CCR7. They also produced high levels of IL-12p70, which could be boosted further by stimulation with CD40 ligand. In addition, 2-day “rapid DCs” induced objective clinical responses in some patients. Dauer *et al* produced DCs under a similar 48-hour “FastDC” protocol in which the monocytes acquired immature DC characteristics by two days of culture, downregulated CD14, increased dextran uptake, and responded to the inflammatory chemokine macrophage inflammatory protein-1α (MIP-1α) [16], [17], [18]. The “FastDC” were compared with mature monocyte-derived DCs generated by a standard 7-day protocol, and were found to be equally potent in priming autologous naive T cells using tetanus toxoid as a model antigen. Therefore, these fast “2-day DCs” offer a very attractive choice for loading with synthetic immunodominant peptides. However, it is currently unknown whether the antigen processing machinery of these fast “2-day DCs” is sufficiently developed to efficiently process and cross-present relevant tumor antigens from complex whole tumor lysates.

The use of whole tumor lysates offers distinct advantages in tumor vaccine preparation. First, all patients are eligible for DC-whole tumor lysate therapy as patients are not selected based on their HLA-A2 status. Second, whole tumor lysate provides a rich array of tumor-associated antigens for both CD4^+^ and CD8^+^ T cells. This is important as the parallel presentation of antigens to both T cell types helps generating stronger primary immune responses, and could prevent the emergence of tumor escape. The presence of CD4^+^ T cell help also promotes long-term CD8^+^ T cell memory [19], [20], [21]. In addition, DCs pulsed with whole tumor lysate have shown enhanced efficacy in cancer patients over DCs loaded with defined tumor-associated peptides or proteins, based on meta-analytical data [22]. Tumor cells can be prepared in numerous ways for DC-based immunotherapy [23]. Ultraviolet B (UVB) irradiation and repeat freeze-thaw cycles are the two most common methods for inducing tumor cell death.

In this study, we sought to evaluate DCs cultured for different lengths of time (i.e. 2 days, 4 days or 7 days) for their abilities to: 1) phagocytose UVB-irradiated tumor cell lysate or freeze-thawed whole tumor cell lysate, 2) produce IL-12p70 and IP-10 after maturation with LPS and IFN-γ, and 3) stimulate tumor-specific IFN-γ responses in an autologous setting using DCs and T cells derived from donors or patients with epithelial ovarian carcinoma (EOC). This is the first study demonstrating that Day-4 myeloid DCs are highly immunogenic and highly capable of priming strong tumor-specific T cells in cancer patients and normal healthy donors. Thus, this study provides important preclinical data for the rapid development of potent whole tumor lysate loaded DC vaccines that are applicable to many tumor types.

## Materials and Methods

### Ethics statement

All healthy donors and ovarian cancer patients are confirmed to have given written informed consent to a tissue and blood procurement study allowing *ex vivo* experimentation, which is approved by the University of Pennsylvania's Office of Regulatory Affairs, Institutional Review Board (IRB).

### Generation of dendritic cells

Fresh monocytes from normal healthy HLA-A2^+^ and HLA-A2^−^ donors, who had given written informed consent to the University of Pennsylvania's Office of Regulatory Affairs, Institutional Review Board (IRB)-approved tissue procurement study, were obtained from the University of Pennsylvania Human Immunology Core Facility and prepared using the RosetteSep® Human Monocyte Enrichment Cocktail kit by negative selection according to the manufacturer's protocol (STEMCELL Technologies Inc., Vancouver, Canada). Purified monocytes were resuspended at 5×10^6^ cells/ml in heat-inactivated fetal bovine serum (FBS) [Invitrogen, Carlsbad, CA, USA] containing 10% DMSO and frozen until required. Frozen elutriated monocytes were obtained from ovarian cancer patients who had given written consent to this IRB-approved protocol. Prior to donating monocytes for this study, these patients had undergone debulking surgery, followed by multiple rounds of chemotherapy. Frozen monocytes from healthy volunteers or ovarian cancer patients were rapidly thawed at 37°C, washed twice with DPBS (Cellgro, Manassas, VA, USA) and resuspended at 1×10^6^ cells/ml in serum-free AIM-V media supplemented with 2 mM L-glutamine, 100 units/ml penicillin, and 100 µg/ml streptomycin (i.e. complete AIM-V media) [clinical grade AIM-V from Invitrogen; all others from Cellgro]. Monocytes were cultured in the presence of 250 IU/ml recombinant human GM-CSF and 125 IU/ml IL-4 [both from PeproTech, Rocky Hill, NJ, USA] for 2 days, 4 days, or 7 days at 37°C, 5% CO_2_ to generate Day-2, Day-4 and Day-7 DCs, respectively. At the end of the culture period, DCs were gently harvested, and the surface expression of CD11c, CD14, HLA-DR, and CD1c on DCs was determined. The DC purity was found to be >98%.

### CD3^+^ T cells

Whole T cell populations from normal healthy HLA-A2^+^ donors were obtained from the University of Pennsylvania Human Immunology Core Facility, where the T cells were prepared using the RosetteSep® Human T cell Enrichment Cocktail kit (STEMCELL Technologies Inc) by negative selection according to the manufacturer's protocol and found to be >98% pure.

### Ovarian cancer patients' peripheral blood leukocytes

Frozen elutriated peripheral blood leukocytes (PBLs) were obtained from ovarian cancer patients who had given written consent to this IRB-approved protocol. Prior to donating PBLs for this study, patients had undergone debulking surgery and multiple rounds of chemotherapy.

### Preparation of UVB-irradiated lysate and freeze-thawed lysate of SKOV3

SKOV3 ovarian carcinoma cells were cultured in DMEM media supplemented with 10% heat-inactivated FBS, 2 mM L-glutamine, 100 units/ml penicillin, and 100 µg/ml streptomycin (FBS from Invitrogen; all others from Cellgro). The cell line was routinely tested for Mycoplasma and found to be negative. To prepare UVB-irradiated and freeze-thaw SKOV3 lysates, 90% confluent SKOV3 cell cultures were harvested, washed twice with DPBS, and resuspended at 1×10^6^ cells/ml in complete AIM-V media. For UVB-irradiation, SKOV3 cells were plated in 10 cm Petri dishes (BD Falcon, San Jose, CA, USA) and subjected to a 302 nm UVB-irradiation (Spectroline, Westbury, NY, USA) for 10 min to induce apoptosis. Then cells were incubated overnight at 37C, 5% CO_2_, and harvested on the following day for 6 cycles of freeze-thaw treatment (freezing with dry ice for 20 min and thawing at room temperature) before use. For freeze-thaw lysate preparation, SKOV3 cells were transferred to 15 ml tubes (BD Falcon) and subjected to 6 cycles of freeze-thaw treatment (as above) before use.

### Dendritic cell phenotyping

Day-2, Day-4, or Day-7 DCs were cocultured with UVB-irradiated or freeze-thawed SKOV3 lysate at a cell ratio of 1∶1 for 16 h at 37°C, 5% CO_2_. Then, 60 EU/ml of LPS (*Escherchia coli*; Sigma, St. Louis, MO, USA) and 2000 IU/ml of IFN-γ (PeproTech) were added to the DC-tumor cocultures for a further 16 h to stimulate DC maturation. Unpulsed Day-2, Day-4, and Day-7 DCs were cultured with or without LPS and IFN-γ and used as mature DCs (mDCs) and immature DCs (iDCs), respectively. After 16 h, the cocultures were harvested and blocked on ice for 10 min with cold staining buffer (DPBS containing 2% heat-inactivated FBS). DCs in cocultures were identified by staining with APC-conjugated anti-HLA-DR, PE-conjugated anti-CD11c, PE-Cy7-conjugated anti-CD14, and one of the FITC-conjugated monoclonal antibodies for DC maturation markers (i.e. CD80, CD86, or CD40), or DC adhesion (ICAM-1) or DC migration (CCR7) for 30 min on ice [all antibodies from BD Pharmingen, San Jose, CA, USA]. For DC-LAMP expression, DCs were stained with FITC-conjugated anti-HLA-DR, PE-conjugated anti-CD1c, and PE-Cy7-conjugated anti-CD14, and then subjected to fix-permeabilization (eBioscience, San Diego, CA, USA) for 30 min at 4°C followed by intracellular staining with APC-conjugated anti-DC LAMP, and finally two washes with staining buffer. Flow cytometry was performed on a BD Canto (Becton Dickinson, Franklin Lakes, NJ, USA) and data were analyzed with Pro CellQuest software. Gated HLA-DR^+^ CD11c^+^ cells were selected for analysis of CD80, CD86, CD40, ICAM-1, CCR7, or DC-LAMP. It was found that >98% of the cells were HLA-DR^+^ CD11c^+^.

### Uptake of UVB-irradiated and freeze-thawed lysates of SKOV3 by dendritic cells

To prepare PKH26-labeled SKOV3 UVB-irradiated or freeze-thaw lysates, tumor cells were first labeled with PKH26 membrane dye (Sigma) according to the manufacturers' protocol and used for lysate preparations as described above. To determine uptake of tumor lysate by Day 2, Day 4, or Day 7 DCs, labeled UVB-irradiated or freeze-thaw SKOV3 lysates were cocultured with DCs at a cell ratio of 1∶1 for 4 h or 24 h at 37°C, 5% CO_2_. Parallel control cultures were set up for 4 h or 24 h at 4°C to evaluate the passive transfer of dye or labeled tumor fragments to DCs. After incubation, cocultures were harvested and DCs identified by staining with APC-conjugated anti-HLA-DR for 30 min on ice. Cells were washed twice with staining buffer and analyzed by flow cytometry as above. HLA-DR^+^ cells were selected for analysis for PKH26 positivity. DCs that had taken up PKH26-labeled SKOV3 lysates were HLA-DR^+^ PKH26^+^.

### ELISA

To evaluate production of IL-12p70 and IP-10 from lysate-pulsed mature Day-2, Day-4, or Day-7 DCs, DCs were cocultured with UVB-irradiated or freeze-thaw SKOV3 lysates at a cell ratio of 1∶1 for 16 h and matured with LPS and IFN-γ for another 12 to 30 h to stimulate DC maturation. Coculture supernatants were then collected by centrifugation for assessment. Unpulsed Day-2, Day-4, or Day-7 DCs were cultured with or without LPS and IFN-γ (mature and immature DCs, respectively, as controls). Briefly, NUNC MaxiSorp™ 96-well ELISA plates (Fisher Scientific, Rochester, NY, USA) were coated with capture IL-12p70 (BioLegend, San Diego, CA, USA) or IP-10 antibody (final concentration of 1.5 ng/ml; BD Pharmingen) for 2 h at 37°C, 5% CO_2_. Plates were then washed 4 times, and appropriately diluted supernatants were incubated overnight at 4°C. Biotinylated detection antibodies for IL-12p70 (BioLegend) or IP-10 (1.5 ng/ml; BD Pharmingen) were added for 1 h incubation at room temperature. Then plates were washed 5 times, and incubated with alkaline phosphatase-conjugated streptavidin for 30 min, and washed again prior to addition of TBM substrate solution. Reactions were stopped by adding equal volumes of 1 N sulfuric acid, and read using an ELISA plate reader (BioTek). A similar protocol (BioLegend) was used to evaluate IFN-γ production from autologous T cells after 2 weeks of stimulation with lysate-pulsed DCs.

### Autologous T cell priming

For analysis of samples from normal healthy HLA-A2^+^ donors, Day-2, Day-4, or Day-7 DCs were cocultured with UVB-irradiated or freeze-thaw SKOV3 lysates at a cell ratio of 1∶1 for 16 h, then matured with LPS and IFN-γ for 16 h, as above. Then 2×10^6^ DCs were harvested, washed twice with DPBS, and cocultured with 2×10^7^ autologous CD3^+^ T cells in complete AIM-V supplemented with 5% human AB serum (Valley Biomedical Inc., Winchester, VA, USA). IL-2 (50 IU/ml) and IL-7 (20 ng/ml) (both from PeproTech) were added on days 3, 5, and 7 of coculture. After 1 week, viable CD3^+^ T cells were harvested, washed twice with DPBS, and cocultured at 10 T cells to 1 DC ratio for another week. Fresh IL-2 and IL-7 were added on days 3, 5 and 7 of the second week of coculture. To determine non-antigen-specific T cell responses, cocultures of unpulsed Day-2, Day-4, or Day-7 DCs and CD3^+^ T cells were set up in parallel. At the end of the second week culture, T cells were harvested, washed, and plated in 96-well round bottom plates with fixed SKOV3 expressing HLA-A2 (i.e. HLA-A2^+^ SKOV3) cells. SKOV3 tumor cells were fixed for 1 min with 0.05% glutaraldehyde (Sigma) and washed twice with DPBS before use. T2 cells (HLA-A2^+^ human T-B lymphoblast hybrids) were pulsed with 10 µg/ml HER-2/neu_369–377_ or HER-2/neu_689–697_ peptides (all peptides ≥95% purity as determined by reverse-phase high performance liquid chromatography; AnaSpec Inc, CA, USA) or media (i.e. T cells alone) for 20 h at 37°C, 5% CO_2_. Then supernatants were harvested and IFN-γ production was assessed by ELISA. For ovarian cancer patient sample analysis, Day-2, Day-4, or Day-7 DCs were generated from autologous elutriated monocytes and cocultured with UVB-irradiated or freeze-thaw SKOV3 lysates at a cell ratio of 1∶1 for 16 h, and matured with LPS and IFN-γ for 16 h, as described above. Then DCs were harvested and cocultured with the patient's autologous PBLs for a total of 2 weeks, using the same procedures described above for healthy volunteer autologous DC-T cell cocultures. At the end of the second week culture, the patients' T cells were harvested, washed, and evaluated in IFN-γ ELISPOT assay.

### IFN-γ ELISPOT

IFN-γ ELISPOT was performed according to the manufacturer's recommendations. Briefly, Multi-Screen™-Immobilon™-P Filtration Plate (Millipore, Bedford, USA) were activated for 30 sec with sterile 70% ethanol (100 µl/well), then washed 5× with sterile distilled water (200 µl/well). Wells were coated with anti-human IFN-γ capture antibody (1-D1K clone; Mabtech, Inc., Mariemont, OH, USA) at 15 µg/ml in DPBS (100 µl/well) overnight at 4°C. Then plates were washed five times with DPBS and blocked with AIM-V media containing 10% human AB serum for 1 h at 37°C, 5% CO_2_. Following that, media was removed and T responder cells were seeded in the wells at 1×10^5^ cells/well. Cryopreserved autologous DCs that had been previously loaded with freeze-thaw SKOV3 lysate (i.e. FTL), and matured with LPS and IFN-γ for 16 h, were rapidly thawed, washed, and fixed for 30 seconds with 0.05% glutaraldehyde before coculturing with T responder cells at a 10 T cells to 1 DC ratio. As specificity controls, T responder cells were cocultured with unloaded mature DCs, or in the presence of media only. HLA-A2^+^ patient T responder cells were also tested for their ability to secrete IFN-γ in the presence of fixed HLA-A2^+^ SKOV3 cells, T2 cells loaded with 10 µg/ml of HER-2/neu_369–377_ or HER-2/neu_689–697_ peptides, or unpulsed T2 cells as background control. The ELISPOT plate was incubated for 40 h at 37°C, 5% CO_2_. After incubation, cells were removed by washing five times with DPBS. The presence of IFN-γ produced by antigen-specific T cells was detected by the sequential addition of biotinylated mouse anti-human IFN-γ (2 h at room temperature), 5 washes with DPBS, alkaline phosphatase-conjugated streptavidin (1 h at room temperature), five further washes with DPBS, and substrates for streptavidin. The number of spots corresponding to the IFN-γ producing cells was counted with an automatic plate reader (Autoimmune Diagnostica GmbH, Strassberg, Germany), and the results were expressed as IFN-γ spots per 10^6^ T cells.

### Statistical methods

**Figure 1:**
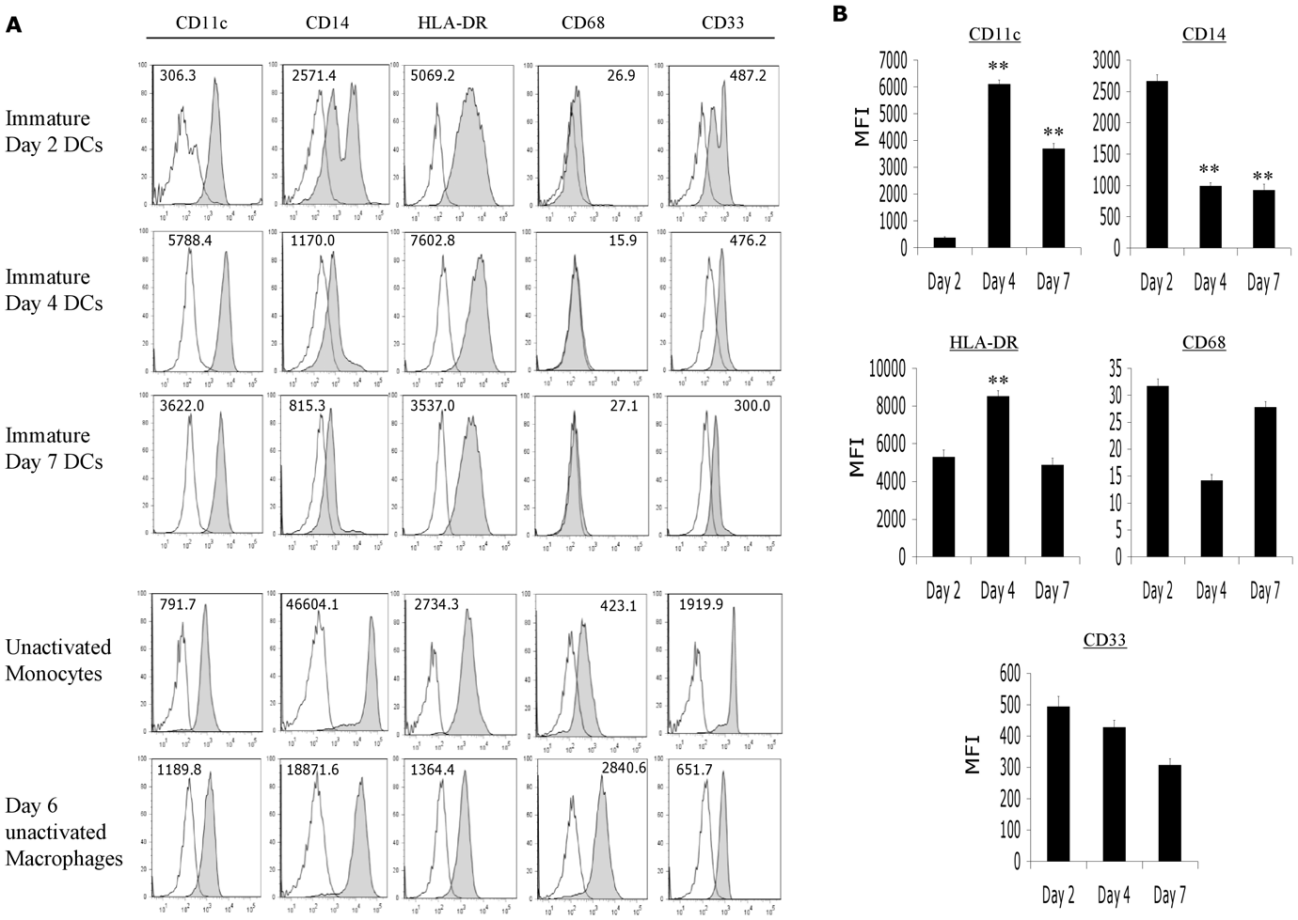
Day-4 and Day-7 DCs, but not Day-2 DCs acquired phenotypic features of differentiated DCs. Elutriated monocytes derived from normal healthy donors were cultured for 2, 4, or 7 days in serum-free AIM-V media supplemented with recombinant GM-CSF and IL-4, and analyzed by flow cytometry at the end of the culture period. (A) Representative histograms from 1 out of 6 donors were shown here. The MFIs indicated in the histograms were derived by subtracting the MFI of the test sample from the isotype control. Day-2 DCs expressed slightly higher CD14, CD68, and CD33 markers than Day-4 and Day-7 DCs, while Day-4 DCs were similar to Day-7 DCs in immunophenotype. (B) The average MFIs of the different DC maturation markers of 6 normal healthy donors were shown here. ** *P* value = <0.001; highly significant when comparing the MFI of Day-4 or Day-7 DCs to the MFI of Day-2 DCs by Student's unpaired *t* test.

**Figure 2:**
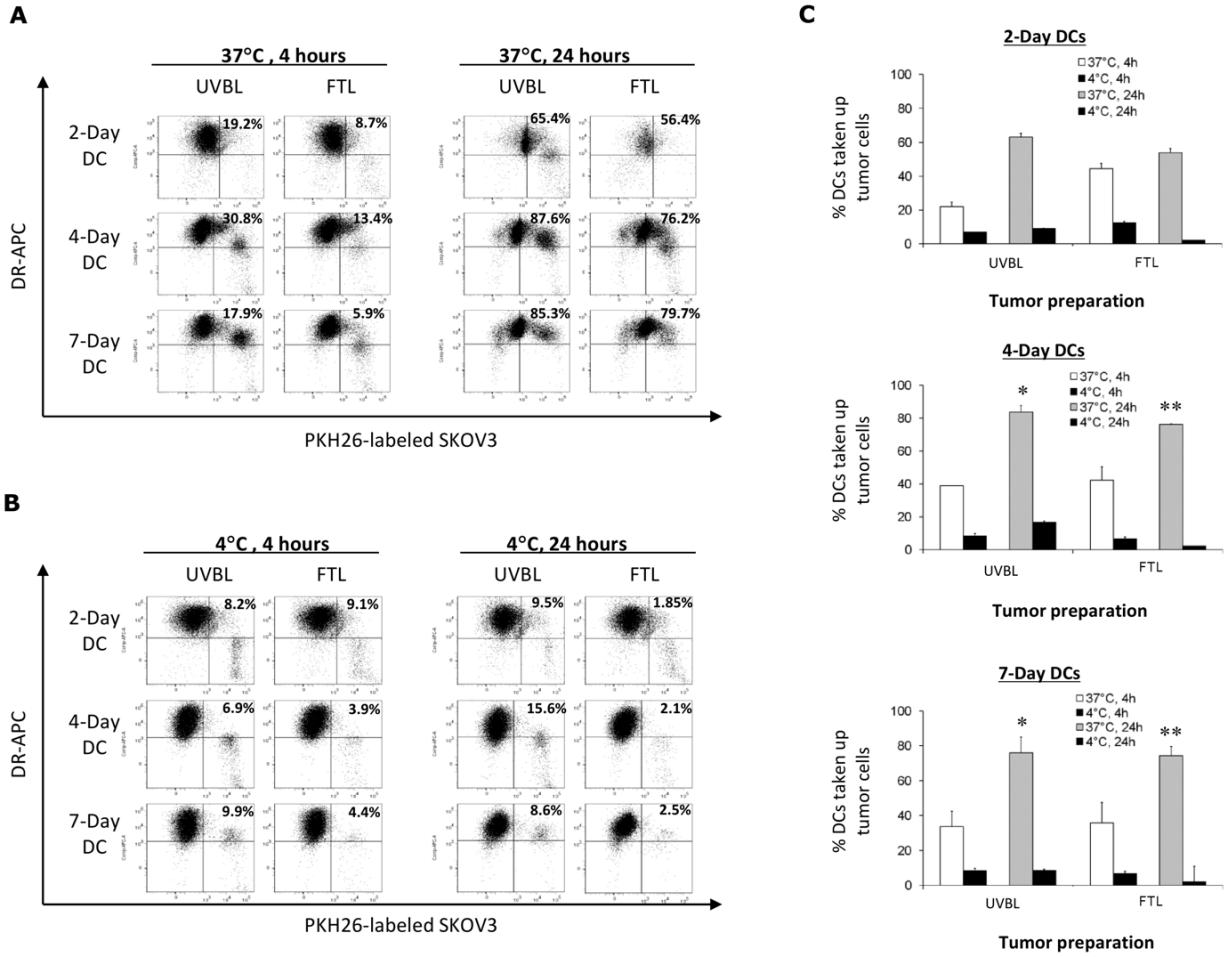
Day-4 DCs, but not Day-2 DCs, were as phagocytic as Day-7 DCs in actively engulfing ovarian tumor lysate. (A) Day-2, Day-4, and Day-7 DCs were cocultured with UVB lysate (UVBL) or freeze-thaw lysate (FTL) of PKH26-labeled SKOV3 cells at 37°C for 4 h or 24 h to determine active phagocytosis of tumor lysates. Representative dot plot results from 1 out of 6 donors are shown here. DCs that had engulfed PKH26-labeled tumor lysate appear as the HLA-DR^+^ PKH26^+^ double-positive population, and are expressed as the percentage of the total number of HLA-DR^+^ DCs as indicated in the upper-right quadrant of the dot plots. (B) DCs were cocultured with lysate at 4°C for 4 h or 24 h as controls to determine passive transfer of the PKH26 dye to DCs. Representative dot plot results from 1 out of 6 donors are shown here. HLA-DR^+^ PKH26^+^ double-positive DCs are expressed as the percentage of the total number of HLA-DR^+^ DCs as indicated in the upper-right quadrant of the dot plots. (C) Summary results of the percentages of Day-2, Day-4, and Day-7 DCs that have engulfed PKH26-labeled UVBL or FTL after 4 h or 24 h, at both 4°C and 37°C. Data displayed are the means ± standard errors of six independent experiments. There are no significant differences among Day-2, Day-4 and Day-7 DCs for the precentage of DCs taking up UVBL (ANOVA *P* value = 0.13) or FTL (ANOVA *P* value = 0.59) at 4 h. However, there is a significant difference for % of DCs taking up UVBL at 24 h (ANOVA *P* value = 0.02). By *post hoc* paired testing, % of Day-2 DCs taking up UVBL was significantly lower than either Day-7 or Day-4 DCs (*P values = 0.05 and 0.02, respectively). Highly significant differences were also observed for the uptake of FTL at 24 h (ANOVA**P value<0.001). The % of Day-2 DCs taking up FTL was significantly lower than either Day-7 or Day-4 DCs (**P values<0.001 for each). The differences between Day-4 and Day-7 DCs were insignificant for uptake of both UVBL (*P* value = 0.90) and FTL (*P* value = 0.92).

**Figure 3:**
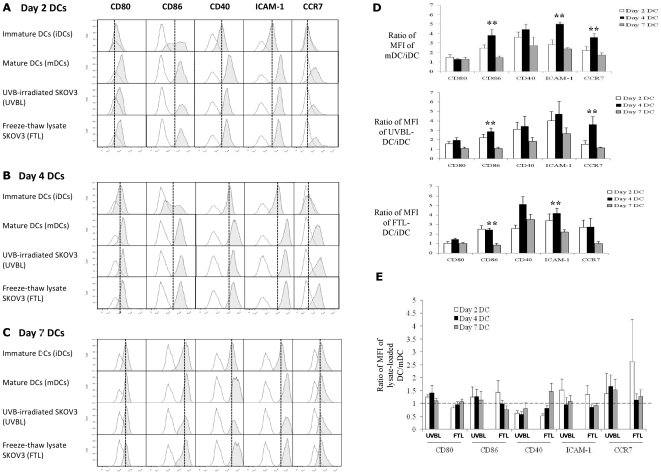
Day-2, Day-4 and Day-7 DCs pulsed with UVBL or FTL mature normally following LPS and IFN-γ stimulation. Following lysate loading, DCs were stimulated with LPS (60 EU/ml) and IFN-γ (2000 IU/ml) for 16 h. DCs were identified by HLA-DR, CD11c and one of the markers shown. Unpulsed immature DCs (iDCs) and mature (mDCs) were set up in parallel and harvested at the same time as the other tumor lysate-loaded mature DCs for analysis. (A–C) Upregulation of maturation markers CD80, CD40, CD86, ICAM-1, and CCR7 is observed on mature Day-2, Day-4 and Day-7 DCs. The open histograms represent the isotype control, while shaded histograms represent the DC markers. The solid line marks the MFI of the different markers on iDCs for each condition. Representative histogram results from 1 out of 6 donors are shown here. (D) Fold-increase in expression levels of maturation and adhesion markers on mDCs, UVBL-loaded DCs (UVBL-DC) or FTL-loaded DCs (FTL-DC) over iDC was determined by expressing the MFIs as a ratio of the DCs to unpulsed iDCs. Highly significant differences were detected in CD86 (**P value = 0.002), ICAM-1 (**P value = <0.001), and CCR7 (**P value = 0.002, <0.001 and 0.008, respectively; ANOVA followed by *post-hoc* testing) on unpulsed matured Day-4 DCs compared to unpulsed matured Day-7 DCs. Similar highly significant differences were detected in CD86 and CCR7 (**P value = 0.001, and 0.01, respectively; ANOVA followed by *post-hoc* testing) on Day-4 UVBL-DCs compared to Day-7 UVBL-DCs. CD86 and ICAM-1 (**P value = 0.001, and 0.01, respectively; ANOVA followed by *post-hoc* testing) were also significantly different on Day-4 FTL-DCs compared to Day-7 FTL-DCs. (E) The overall immunophenotypes of DCs loaded with UVBL or FTL were similar to unpulsed mature DCs, however Day-4 DCs loaded with UVBL consistently expressed slightly higher levels of most markers including CD80, CD86 and CCR7 relative to any other lysate-DC preparations.

**Figure 4:**
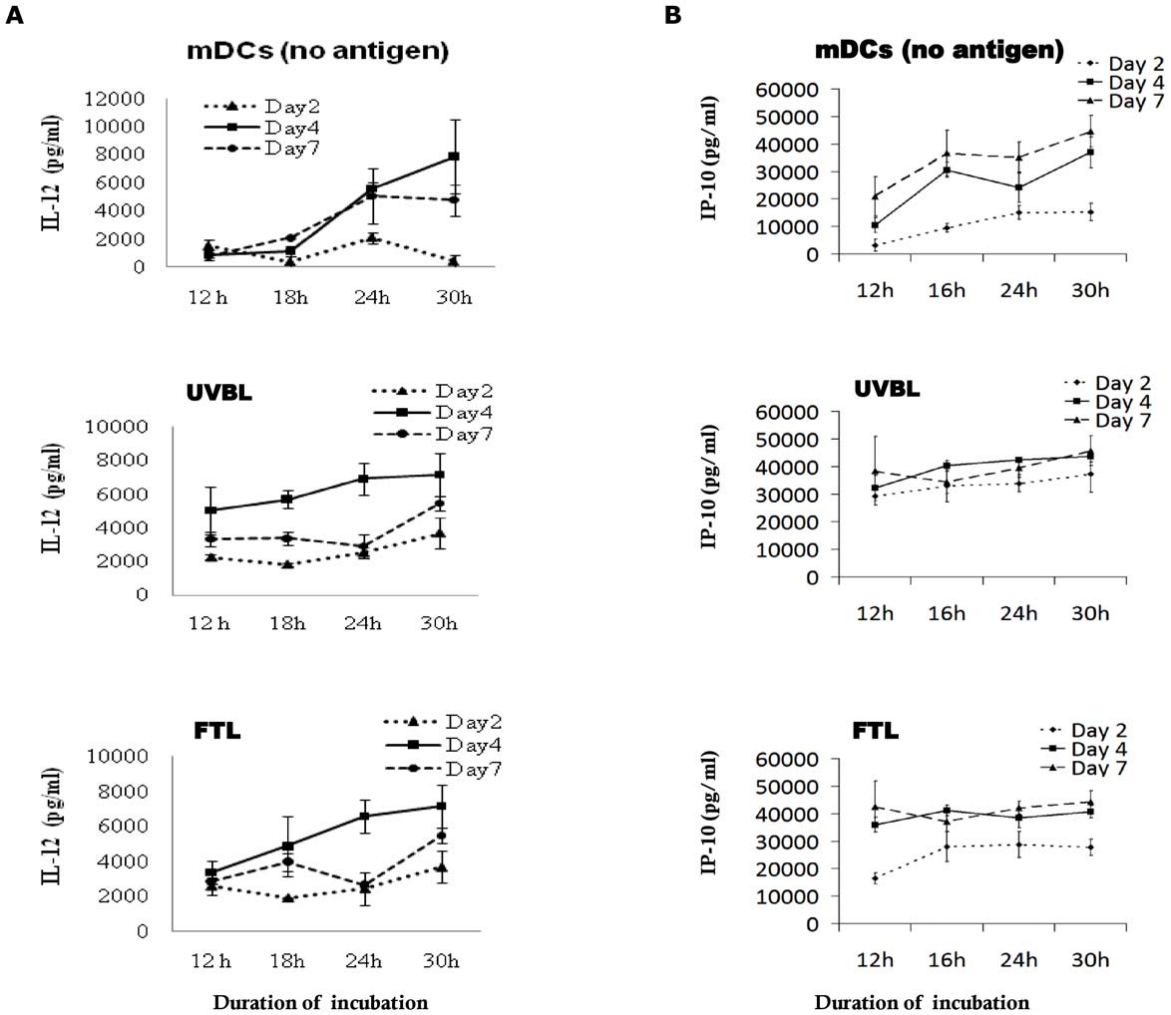
Day-4 DCs produce the highest levels of IL-12p70 and IP-10. Day-2, Day-4, and Day-7 DCs were first pulsed with either UVBL or FTL for 16 h, and then stimulated with LPS (60 EU/ml) and IFN-γ (2000 IU/ml) to determine (A) IL-12p70 and, (B) IP-10 production. Supernatants from the DC-tumor lysate cocultures were collected and evaluated by ELISA as described in Materials and Methods. The results are from 4 different normal healthy donors and are expressed as mean (pg/ml) ± standard errors.

**Figure 5:**
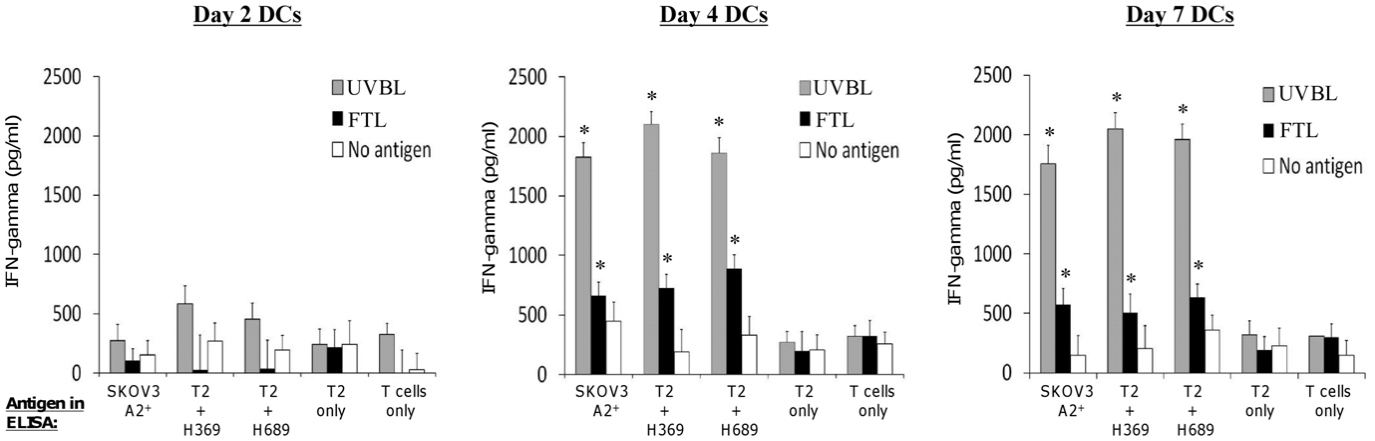
Day-4 DCs derived from normal healthy HLA-A2^+^ donors pulsed with UVBL or FTL and matured with LPS and IFN-γ, stimulate potent and specific autologous ovarian tumor-reactive T cell responses *in vitro*. Day-2, Day-4, and Day-7 DCs were pulsed with lysate for 16 h and matured with LPS (60 EU/ml) and IFN-γ (2000 IU/ml) for a further 16 h before being used for priming T cells. After 2 weeks of *in vitro* stimulation, T cells were harvested and evaluated for their ability to recognize ovarian tumor-associated antigens. The IFN-γ responses of T cells previously stimulated with UVBL-pulsed DCs (grey bars), FTL-pulsed DCs (black bars) or unpulsed mature [no antigen] DCs (white bars) are shown here. Data were obtained from 3 different individuals and displayed as the means ± standard errors. Day-2 DCs result in no significant T cell priming, while Day-4 and Day-7 DCs elicit T cells that are able to produce significant amounts of IFN-γ in response to live HLA-A2^+^ SKOV3 cells or to HLA-A2^+^ T2 cells pulsed with HER-2/neu_369–377_ (H369) or HER-2/neu_689–697_ (H689). Using Kruskal-Wallis test by *post-hoc* paired testing, Day-2 DCs are significantly lower than either Day-7 or Day-4 DCs (*P values = 0.05 for all tests), and no differences are detected when comparing Day-4 and Day-7 DCs (*P* values ≥ 0.28). FTL-pulsed DCs perform better than UVBL-pulsed DCs in priming ovarian-specific IFN-γ T cell responses.

**Figure 6:**
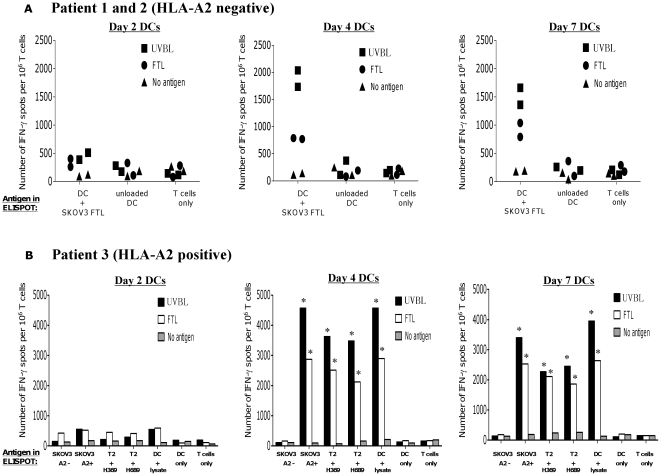
Day-4 DCs derived from EOC patients, stimulated potent and specific autologous ovarian tumor-reactive T cell responses *in vitro*. Patients' T cells were cocultured with autologous Day-2, Day-4 or Day-7 mature DCs previously pulsed with SKOV3 UVBL or FTL for 2 weeks and then interrogated for reactivity against tumor antigens. The IFN-γ responses of patients' T cells previously stimulated with UVBL-pulsed DCs (closed squares or black bars), FTL-pulsed DCs (closed circles or white bars) or unpulsed mature DCs (closed triangles or grey bars) are shown here, (A) For patients 1 and 2 who were HLA-A2^−^, the T cells were tested in the presence of autologous DCs pulsed with SKOV3 FTL, autologous unpulsed DCs, or media (T cells only). (B) For patient 3, who was HLA-A2^+^, the T cells were also tested against live HLA-A2^+^ SKOV3 cells, HLA-A2^+^ T2 pulsed with HER-2/neu 369 or 689 peptides, autologous DCs pulsed with HLA-A2^−^ SKOV3 FTL, autologous unpulsed DCs, or media (T cells only). The results are the means of the number of IFN-γ spots per 10^6^ T cells ± standard error. The asterisks indicate those columns differing significantly (*P* value = 0.001; Student's paired *t* test) from the media (i.e. T cells only) controls.

Measurements taken for Day-2, Day-4, and Day-7 DCs were summarized by means and standard errors. For figure 1, statistical significance was assessed using Student's unpaired *t* test. The comparisons among groups for % DC uptake of tumor cells in figure 2, and the ratios of mature DC (mDC), UVB-lysate loaded DC (UVB-DC) and FTL-loaded DC (FTL-DC) to immature DC (iDC), and mean fluorescence intensity (MFI) of mDCs in figure 3, were performed on natural log transformed data using ANOVA. For those comparisons found to be statistically significant by ANOVA, Tukey *post hoc* testing was used to conduct pairwise comparisons. Measurements in figure 4 were summarized by means and standard errors. In figure 5, IFN-γ measurements were compared amongst groups by the nonparametric Kruskal-Wallis test due to the small group sizes and non-normality of the data, and pairwise comparisons were performed by Wilcoxon rank sum testing. In figure 6, statistical significance was assessed using Student's paired *t* test. A significance level of 0.05 or less was considered statistically significant. Analyses were performed in SPSS v19 (SPSS Inc, Chicago, IL).

## Results

### Phenotype of Day-2, Day-4 and Day-7 immature dendritic cells

As monocytes differentiate into immature dendritic cells (iDCs) in the presence of recombinant GM-CSF and IL-4, they upregulate CD11c, lose CD14 surface expression and develop distinct dendritic appearance. To evaluate iDCs for CD11c, CD14, HLA-DR, CD68, and CD33 expression and morphology, elutriated monocytes were cultured in serum-free AIM-V media in the presence of GM-CSF (250 IU/ml) and IL-4 (125 IU/ml) for 2 days, 4 days or 7 days to induce differentiation into iDCs. It was found that 100% of unpulsed Day-2, Day-4, or Day-7 iDCs expressed CD11c. However, Day-2 iDCs expressed approximately 20- and 10-fold lower CD11c compared to Day-4 iDCs (*P* = <0.0001) and Day-7 iDCs (*P* = <0.0001) [Figs. 1A, B]. All three DC preparations had substantially lower CD14 expression compared to starting monocytes and Day-6 macrophages that were developed only with macrophage colony-stimulating factor (Figs. 1A, B). The lowest CD14 expression was found on Day-4 and Day-7 iDCs (*P* = <0.0001), while Day-2 iDCs comprised a population with higher CD14 expression. All three DC preparations expressed intermediate levels of HLA-DR, as expected of iDCs. However, Day-4 iDCs expressed the highest level of HLA-DR (*P* values = <0.0002; compared to Day 2 and Day 7 iDCs). All iDCs exhibited low CD33 and CD68, which are markers for monocytes and macrophages, respectively (Figs. 1A, B). Day-2 iDCs were smaller in size as compared to Day-4 and Day-7 iDCs by flow cytometry analysis (data not shown). Thus, Day-4 iDCs were phenotypically similar to Day-7 iDCs, while Day-2 iDCs were not fully differentiated.

### Phagocytic ability of Day 2, Day 4 and Day 7 immature dendritic cells

To prepare tumor cell lysates, SKOV3 tumor cells were labeled with PKH26 and treated with 1) UVB-irradiation, followed by 6 cycles of freeze-thaw to generate UVB-whole tumor cell lysate (UVBL), or 2) 6 cycles of freeze-thaw alone to generate freeze-thawed whole tumor cell lysate (FTL). To assess the phagocytic ability of immature Day-2, Day-4 and Day-7 DCs, UVBL or FTL lysate preparations were then cocultured with the different DC preparations. DCs were identified by the expression of HLA-DR, and DCs that had phagocytosed tumor lysate were identified as HLA-DR^+^ PKH26^+^ double-positive. There was a rapid uptake of UVBL by Day-2, Day-4, and Day-7 iDCs at 37°C after 4 h of incubation (Fig. 2A). Day-4 and Day-7 iDCs showed similarly strong phagocytic capabilities at 24 h at 37°C, whereas Day-2 iDCs took up less tumor lysate under the same conditions (Fig. 2A). Phagocytosis of FTL occurred to a slightly lesser extent than UVBL after 24 h at 37°C. (Fig. 2A). Uptake of tumor lysate by the iDCs was confirmed to be an active process, as passive transfer of tumor fragments onto DC surfaces at 4°C was minimal (Fig. 2B). Summarizing the results from 6 normal healthy donor DC samples in Fig. 2C, Day-4 and Day-7 iDCs showed similarly high uptake (≥75%) of UVBL and FTL after 24 h at 37°C [*P* = 0.90 and p = 0.92, respectively], and Day-2 iDCs showed a significantly lower uptake ability at 24 h at 37°C (approximately 50–60%; *P* = 0.05 for UVBL uptake and <0.0001 for FTL uptake when compared to Day-4 and Day-7 iDCs). Thus, Day-4 iDCs were as capable as Day-7 iDCs in the phagocytosis of whole tumor cell lysates.

### Maturation of tumor lysate-pulsed Day 2, Day 4, and Day 7 dendritic cells

One of the potential concerns of preparing lysate-loaded DCs is that tumor lysates could suppress the ability of DCs to undergo proper maturation. To investigate whether Day-2, Day-4 or Day-7 iDCs that had phagocytosed UVBL or FTL were able to respond normally to LPS and IFN-γ stimulation, DCs were incubated with the relevant lysate for 16 h and stimulated with 60 EU/ml LPS and 2000 IU/ml IFN-γ for an additional 16 h. Lysate-loaded Day-2 and Day-4 iDCs upregulated maturation and migration markers upon stimulation with LPS and IFN-γ when compared to their non-stimulated, unpulsed counterparts (Figs. 3A, B). Non-stimulated, unpulsed immature Day-7 DCs showed upregulation of markers such as CD80, CD40 and CD86 (Fig. 3C, top panel) in serum-free culture conditions, in agreement with other groups [24], [25], [26]. Nevertheless, Day-7 lysate-loaded iDCs were able to further upregulate CD40 and ICAM-1 expression upon stimulation with LPS and IFN-γ. Summary results from 6 different donors are shown in Fig. 3D; expression changes are shown as fold-upregulation of mean fluorescence intensity (MFI) following exposure to LPS and IFN-γ over baseline (non-stimulated and unpulsed) iDCs at the same time point. Numerous markers were significantly upregulated over baseline under various conditions (Fig. 3D). Day-4 DCs, whether unpulsed and matured, or pulsed with UVBL or FTL and matured, showed higher relative upregulation of most markers (except CD80 when loaded with FTL) in comparison to Day-7 DCs. Day-7 DCs that were pulsed with lysate and stimulated with LPS and IFN-γ showed no further upregulation of CD80, CD86 or CCR7 over baseline Day-7 DCs.

To evaluate the final immunophenotype of mature Day-2, Day-4 and Day-7 DCs pulsed with either UVBL or FTL, we compared them to mature unpulsed DCs. Overall, the immunophenotypes of mature DCs pulsed with FTL or UVBL were similar to unpulsed mature DCs, with some exceptions (Fig. 3E). Day-2, Day-4 and Day-7 UVBL-pulsed mature DCs showed lower CD40 than mature unpulsed DCs. This was also the case with Day-2 and Day-4 FTL-pulsed mature DCs, but CD40 was upregulated in Day-7 FTL-pulsed mature DCs. Interestingly, Day-2 and Day-4 DCs pulsed with UVBL had overall a very similar immunophenotype after maturation with LPS and IFN-γ. Upon analysis of all the surface markers, it was found that Day-4 DCs pulsed with UVBL consistently expressed slightly higher levels of most markers including CD80, CD86 and CCR7 relative to any other lysate-DC preparation.

### IL-12p70 and IP-10 production by lysate-pulsed dendritic cells

IL-12 produced by DCs is critical for complete priming of naive T cells. To determine the IL-12p70 production profile of Day-2, Day-4 and Day-7 DCs, DCs were first pulsed with either UVBL or FTL for 16 h, and then stimulated with LPS and IFN-γ for 12 to 30 h. IL-12p70 protein was quantified in culture supernatants at 12, 18, 24, or 30 h. Day-2 UVBL or FTL-pulsed DCs that were stimulated with LPS and IFN-γ produced the least amount of IL-12p70 at 30 h (Fig. 4A). Day-4 DCs pulsed with either UVBL or FTL produced higher amount of IL-12p70 than did lysate-pulsed Day-2 or Day-7 DCs after LPS and IFN-γ stimulation. IL-12p70 production increased steadily through 30 h (Fig. 4A). As expected, unpulsed and unstimulated Day-2, Day-4 and Day-7 DCs (no LPS or IFN-γ) did not produce IL-12p70 (data not shown). Importantly, Day-2, Day-4 and Day-7 DCs that had phagocytosed tumor fragments did not exhibit impaired IL-12p70 production after LPS and IFN-γ stimulation, for they displayed similar profiles as their unpulsed mature Day-4 or Day-7 counterparts [Fig. 4A; DC (no antigen)]. Interestingly, lysate-pulsed mature Day-2 DCs produced higher IL-12p70 compared to their unpulsed mature counterparts.

Interferon-inducible protein (IP)-10 (also known as CXCL10) chemokine is produced by many cell types including DCs. Its expression is induced by IFN-γ and TNF-α, and it is used as an indication of IFN-γ activity [27]. It was noted that IP-10 production by UVBL-pulsed and FTL-pulsed DCs started early, with high levels already being produced at 12 h. UVBL-pulsed mature Day-2, Day-4, and Day-7 DCs produced similarly high amount of IP-10 at 30 h (Fig. 4B). For FTL-pulsed DCs, Day-4 and Day-7 DCs produced similar IP-10, whereas Day-2 DCs produced less IP-10 at 30 h. Mature unpulsed DCs followed a similar pattern of IP-10 production as FTL-lysate pulsed DCs, with Day-4 and Day-7 DCs producing more IP-10 than Day-2 DCs (Fig. 4B). In summary, Day-4 UVBL-pulsed mature DCs produced the highest amount of IL-12p70 and had comparable expression of IP-10 to all other lysate-DC preparations.

### Priming of tumor-reactive T cells by autologous tumor lysate-pulsed matured dendritic cells

To assess the ability of Day-2, Day-4, and Day-7 DCs to prime human donor T cells reactive against tumor antigens, DCs developed from the peripheral blood mononuclear cells (PBMCs) of normal healthy HLA-A2^+^ donor as above were either left unpulsed or pulsed with UVBL or FTL of SKOV3 cells, then matured with LPS and IFN-γ, and used to stimulate *in vitro* autologous CD3^+^ T cells for two consecutive weeks. Then, viable autologous T cells were harvested, and evaluated in ELISA for their recall ability to recognize ovarian antigens and produce IFN-γ in response to live HLA-A2^+^ SKOV3 cells. Because SKOV3 cells express HER-2/neu [28], [29], [30], we also tested the ability of DCs to prime donor T cells against HLA-A2 restricted peptides HER-2/neu_369–377_ and HER-2/neu_689–697_ pulsed on T2 cells (which are HLA-A2^+^). Day-4 and Day-7 UVBL-pulsed DCs elicited autologous T cells that secreted high IFN-γ against HLA-A2^+^ SKOV3 and T2 cells pulsed with HER-2/neu_369–377_ or HER-2/neu_689–697_ peptides (Fig. 5). In contrast, minimal IFN-γ production was observed when T cells were exposed to unpulsed T2 cells alone or to media (i.e. T cells only; no antigen), indicating that IFN-γ production was ovarian tumor-specific. Day-4 DCs were as potent as Day-7 DCs in priming tumor-reactive T cells to secret high levels of IFN-γ in response to live HLA-A2^+^ SKOV3, and to T2 cells pulsed with HER-2/neu_369–377_ or HER-2/neu_689–697_ peptides. On the other hand, Day-2 DCs stimulated poor tumor-specific IFN-γ production from the T cells (Fig. 5). Importantly, UVBL-pulsed DCs were more potent than FTL-pulsed DCs in eliciting autologous T cells that were reactive to live HLA-A2^+^ SKOV3 and T2 cells pulsed with HER-2/neu peptides. Thus, UVBL-pulsed DCs were more effective in priming tumor-reactive autologous T cells than FTL-pulsed DCs, and Day-4 DCs were as efficient as Day-7 DCs.

Next, we compared the stimulatory capacity of Day-2, Day-4, and Day-7 DCs derived from elutriated monocytes of three ovarian cancer patients to elicit tumor-specific T cell responses. DCs generated from the patients were pulsed with SKOV3-derived UVBL or FTL and evaluated for their ability to stimulate antigen-specific T cell responses. After two weeks of stimulation with lysate-pulsed DCs or unpulsed DCs, bulk autologous T cells were harvested for IFN-γ ELISPOT. T cells were examined for antigen-specific IFN-γ responses by restimulation with DC pulsed with SKOV3 FTL, or unpulsed DCs. For patient 3 who was HLA-A2 positive, T cells were also tested against T2 cells pulsed with HER-2/neu_369_ or HER-2/neu_689_ peptides, and live HLA-A2^+^ SKOV3. T cells alone and T cells stimulated with anti-CD3 antibody (data not shown) were used as control.

T cells from patients 1 and 2 (both were HLA-A2^−^) that had received primary stimulation with FTL-pulsed Day-4 or Day-7 DCs responded to FTL-pulsed DCs in the IFN-γ ELISPOT assay. It was observed that Day-7 FTL-pulsed DCs stimulated a slightly higher number of IFN-γ secreting T cells (average of 900 IFN-γ spots) than did Day-4 FTL-pulsed DCs (average of 750 IFN-γ spots) [Fig. 6A]. UVBL-pulsed Day-4 or Day-7 DCs stimulated a higher number of IFN-γ secreting T cells compared to FTL-pulsed Day-4 or Day-7 DCs. The antigen-specificity of these responses was demonstrated by the low number of IFN-γ secreting T cells (average of 250 IFN-γ spots) when they were cultured alone, or in the presence of Day-4 or Day-7 unpulsed DCs. Day-2 DCs elicited no tumor-reactive IFN-γ producing T cells. Similar results were observed in the T cells of patient 3 who was HLA-A2^+^, where a greater number of T cells that had been previously stimulated with Day-4 or Day-7 UVBL-pulsed DCs produced IFN-γ upon recognition of ovarian antigens as compared to T cells that were previously stimulated with Day-4 or Day-7 FTL-pulsed DCs (Fig. 6B). Day-2 DCs from patient 3 were also unable to elicit anti-tumor responses in the T cells. In addition, a greater number of T cells that were previously stimulated with Day-4 UVBL-pulsed DCs could specifically recognize HER-2/neu_369_ and HER-2/neu_689_ peptides on T2 cells and DCs loaded with SKOV3 FTL compared to T cell that were previously stimulated with Day-7 UVBL-pulsed DCs. These T cells also recognized ovarian HLA-A2^+^ SKOV3. Importantly, very few IFN-γ spots were detected in T cells alone culture or when T cells were cultured with unpulsed DCs in the ELISPOT assay. The IFN-γ ELSIPOT results showed that Day-4 DCs were as potent as Day-7 DCs in eliciting anti-tumor responses in autologous T cells, while Day-2 DCs were incapable of eliciting tumor-reactive IFN-γ T cell responses (Figs. 6A, B).

## Discussion

Human DCs can be differentiated from various cellular sources, such as CD34^+^ progenitor cells from bone marrow and cord blood [31], [32], or circulating monocytes from the PBMC population [9], [10], [11]. As monocytes can easily be obtained in large numbers from peripheral blood, they are currently the most popular precursor cells for generating DCs. In the classic Day-7 DC protocol, monocytes are cultured in the presence of recombinant GM-CSF and IL-4 for a total of 7 days in order to fully differentiate them into myeloid DCs. In our hands, Day-7 monocyte derived DCs markedly downregulated CD14 and CD33 expression, and upregulated CD11c and HLA-DR expression as compared to monocytes or macrophages, in agreement with existing literature. Indeed, Day-7 MoDCs loaded with tumor antigens have already been used successfully in the clinic to elicit anti-tumor responses in cancer patients [1], [2], [3], [4], [5], [6], [7], [8].

To speed DC vaccine preparation, and to reduce costs and labor requirements, shorter Day-2 DC preparation protocols have been developed. These monocyte-derived “rapid DCs” are shown to be similar in phenotype to Day-7 DCs in terms of expressing high levels of CD86, HLA-DR, and CCR7 upon maturation. These Day-2 DCs were also shown to be able to produce IL-12, take up latex beads or dextran [16], [17], [18], and stimulate tetanus toxoid (TT) [17] or anti-HER-2/neu [15] T cell responses when loaded with TT or HER-2/neu peptides. In this study, we sought to optimize the length of DC culture (i.e. 2, 4 or 7 days of culture) for use with whole tumor cell lysate as source of antigens in the clinic. Whole tumor lysates offer many distinct advantages over defined peptides or proteins; for example, T cell responses can be elicited against tumor-associated antigens without prior knowledge of the specific antigens. It is important to note that increased efficacy has been reported when whole tumor lysates were used as a source of antigen in DC-based cancer immunotherapy [23]. Thus, whole tumor lysates are ideal sources of antigens for DCs to prime anti-tumor responses. The use of whole tumor lysate requires DCs to have fully functional antigen processing machinery, to allow processing of complex tumor lysates into peptides for presentation to T cells in association with appropriate MHC molecules. However, it was unknown if Day-2 MoDCs already acquired the necessary antigen processing and presentation machinery for processing whole tumor lysates, and for priming anti-tumor T cells responses.

In order to develop a DC protocol compatible with clinical use, we made the choice of culturing the monocyte-derived DCs in clinial grade serum-free AIM-V media for several reasons: 1) AIM-V media has been widely used for DC and T cell generation; 2) it is approved for clinical use by the Food and Drug Administration (FDA); and 3) it is manufactured under current Good Manufacturing Practices (GMP). Thus, in this regard, our cultures differ from those published by the Dauer [17] and Czerniecki groups ([15], in that we did not use macrophage serum-free media because it is used mainly for growing monocytes and macrophages, and it is not available in therapeutic grade for clinical use. Similarly, we did not use RPMI 1640 media, because although it is widely used for immunological studies including the culturing of DCs, it is only available in research grade not suited for clinical use.

We demonstrated that DCs that were cultured in serum-free AIM-V media in the presence of recombinant GM-CSF and IL-4 for 2 days retained many monocytic-like features, i.e. higher CD14 expression and smaller size compared to Day-7 DCs that were cultured in the same media condition but for 7 days. Although CD14 expression was downregulated after 2 days of DC culture, approximately half of the Day-2 DCs still expressed high levels of surface CD14. These Day-2 DCs also expressed lower levels of CD80, CD86, CD40, ICAM-1 and CCR7 than Day-7 DCs after stimulation with LPS and IFN-γ. It is to be emphasized that monocytes that have been activated with LPS and IFN-γ also upregulate CD86, HLA-DR, and CCR7 expression, and produce high levels of IL-12 [33] in a fashion similar to DCs (data not shown). Therefore, upregulation of these markers in the presence of high levels of CD14 and/or CD33 should not be considered as confirmation of attaining full DC differentiation and maturation. Although Day-2 ‘rapid DCs’ have been demonstrated to be able to stimulate antigen-specific response in breast cancer patients when loaded with tumor-associated peptides [15], no antigen processing is necessary under these circumstances, since the immunodominant peptides can bind directly to the MHC molecules that are expressed on the DCs. Therefore, this could explain why loading MHC Class I-restricted immunodominant peptides on high MHC Class I expressing Day-2 “rapid DCs” can elicit strong peptide-specific CD8^+^ responses in the study conducted by Czerniecki and colleagues.

By extending the monocyte-derived DC culture to 4 days in our culture system, all DCs reduced their CD14 to a level that was comparable to that of Day-7 DCs. This demonstrated that the 4 days of culture was sufficient to differentiate monocytes into DCs that were similar to Day-7 DCs. By day 4, immature DCs had an active phagocytic ability and were as capable as Day-7 iDCs to take up whole tumor cell lysates. Day-4 iDCs were able to upregulate maturation markers such as CD80, CD86, CD40 and ICAM-1, and migration markers such as CCR7, upon LPS and IFN-γ stimulation. In addition, Day-4 iDCs had a higher capacity to upregulate these markers compared to Day-7 iDCs (Fig. 3D). Day-7 iDCs generated with our serum-free protocol exhibited a more mature phenotype compared to Day-2 or Day-4 DCs prior to stimulation with LPS and IFN-γ, with higher CD80, CD86, and HLA-DR expression. However, these Day-7 iDCs exhibited a high phagocytic ability, which is a distinct feature of immature DCs. Also, Day-7 iDCs that were not stimulated with LPS and IFN-γ did not produce IL-12p70 [data not shown], and they only did so after full activation from LPS and IFN-γ stimulation [Fig. 4A]. It should be noted that we initially followed Czerniecki group's protocol [15] by stimulating DCs first with LPS for 6 hours before adding IFN-γ for another 12 hours. However, we could not detect any IL-12p70 production by this staggered manner in our DC culture system (data not shown). When we added both LPS and IFN-gamma simultaneously in the DC cultures, we could consistently detect high levels of IL-12p70. Therefore we chose to administer LPS and IFN-gamma at the same time to mature DCs in this study.

IL-12 is an important cytokine produced by mature DCs upon appropriate simulation, such as bacterial LPS. IL-12 is necessary for skewing towards a Th1 response, which is essential for tumor killing. IFN-γ has a powerful effect in enhancing the ability of DCs to produce IL-12, and IL-12 also enhances the production of IFN-γ, thus creating a positive reinforcement loop [34]. Thus, it was predicted that simultaneous stimulation of DCs with LPS and IFN-γ would induce robust IL-12 production from DCs. IP-10 is a highly inducible, primary response gene that belongs to the C-X-C chemokine superfamily [27], [35]. Its production is increased following IFN-γ stimulation. It has numerous biological actions, including stimulation of monocytes and natural killer cells, T cell migration, regulation of T cell and bone marrow progenitor maturation, modulation of adhesion molecule expression, and inhibition of angiogenesis [35].

We found that in our DC culture system, Day-4 DCs pulsed with either tumor lysate produced higher levels of IL-12p70 at 30 h than did Day-7 or Day-2 DCs. This indicated that Day-4 DCs were equally, if not more, sensitive to LPS and IFN-γ than were Day-2 and Day-7 DCs; their production of IL-12p70 was greater than Day-7 DCs and their IP-10 secretion up to 30 h was comparable to that of Day-7 DCs. In contrast, Day-2 DCs showed the poorest IL-12p70 and IP-10 production, which highly suggested that these DCs had not developed sufficiently to fully respond to LPS and IFN-γ stimulation. It has been demonstrated that DCs that are further stimulated with CD40 ligand after LPS and IFN-γ treatment enhanced their IL-12p70 production [15] and this approach could further enhance the immunogenicity of the Day-4 DCs.

Day-4 lysate pulsed DCs were as potent as Day-7 DCs in stimulating autologous T cells in both healthy donors as well as in ovarian cancer patients; these T cells recognized live ovarian tumor cell lines and ovarian tumor-associated antigens, and produced IFN-γ as a result. Although Day-4 DCs generally expressed lower levels of maturation markers (i.e. CD80 and CD40), and adhesion (ICAM-1) or migration molecules (CCR7) after maturation than Day-7 DCs, this did not affect their ability to stimulate potent ovarian-specific responses in the T cells derived from healthy volunteers and ovarian cancer patients. It was interesting to note that Day-4 or Day-7 DCs that were loaded with UVBL were able to elicit stronger IFN-γ ovarian tumor specific responses compared to DCs loaded with FTL. Different whole tumor cell lysate preparations could potentially impact the immunogenicity of DC-whole tumor lysate vaccines. It could be postulated that mixed apoptotic/necrotic tumor cell death induced by UVB-irradiation, and not cell death via freeze-thawed cycles, was able to release more potent danger signals (e.g. from the release of heat-shock proteins, uric acid or High Mobile group protein B1) for activating DCs. We are currently evaluating and optimizing whole tumor lysate preparations for loading onto these Day-4 myeloid DCs.

In summary, we developed a faster, Day-4 DC protocol using GM-CSF, IL-4, and serum-free AIM-V media that is suitable for clinical use. We showed specifically that Day-4 DCs generated from this protocol are similar to “classic” Day-7 DCs in terms of phenotype and phagocytic capability and have a higher capacity than Day-7 DCs to produce IL-12p70 and IP-10 following LPS and IFN-γ stimulation. In addition, these Day-4 DCs are highly immunogenic, and efficiently prime and stimulate strong ovarian tumor-specific T cells derived from both healthy volunteers and ovarian cancer patients. It should be noted that we did not directly compare the Day-4 DCs generated with this protocol to the Day-2 “rapid DCs” as reported by Dauer *et al.*, or Czerniecki *et al.* [15], [17]. Due to some variations in our method reported here relative to theirs (e.g. media and maturation stimuli for DCs), we could not conclude that Day-4 DCs are universally superior to Day-2 DCs in all culture systems and for all antigen types. However, based on the results in this study, we conclude that Day-4 DCs generated using GM-CSF, IL-4 and serum-free AIM-V media and pulsed with whole tumor antigen are superior to Day-2 DCs generated in the same culture condition for priming strong and specific tumor-specific T cell responses. This is the first study demonstrating the strong T cell stimulatory capability of shorter Day-4 DCs pulsed with whole tumor lysate. Given the overall superior performance of whole-tumor lysate preparations over molecularly defined antigens for cancer vaccines and the overall superiority of DC-based vaccines [22], our results provide important preclinical data for the rapid development of potent, highly immunogenic DC-whole tumor lysate vaccines for treating many tumor types.
